# Nanomaterials reshape the pulmonary mechanical microenvironment: novel therapeutic strategies for respiratory diseases

**DOI:** 10.3389/fbioe.2025.1597387

**Published:** 2025-05-02

**Authors:** Li-zhen Chen, Peng-fei Zheng, Qi Cai, Run-nan Chen

**Affiliations:** ^1^ Department of Pharmacy, The First Hospital of Putian City, Putian, Fujian, China; ^2^ College of Environmental and Biological Engineering, Putian University, Fujian, China; ^3^ School of Computer Science and Technology, University of Science and Technology of China, Hefei, Anhui, China; ^4^ School of Medicine, Nankai University, Tianjin, China; ^5^ Department of Respiratory and Critical Illness Medicine, The First Hospital of Putian, Putian, Fujian, China

**Keywords:** nanomaterials, respiratory diseases, pulmonary mechanical microenvironment, drug delivery, chronic obstructive pulmonary disease, stimuli-responsive materials, extracellular matrix

## Abstract

Respiratory diseases, including chronic obstructive pulmonary disease (COPD), idiopathic pulmonary fibrosis (IPF), and lung cancer, exhibit elevated death rates and pathological intricacy, requiring advancements that surpass the constraints of traditional therapies. This study comprehensively outlines the novel applications of nanomaterials in respiratory medicine by accurately modulating the pulmonary mechanical microenvironment, encompassing alveolar surface tension, extracellular matrix rigidity, and the immune-fibroblast interaction network. The precise delivery, stimuli-responsive characteristics, and biomimetic design of nanomaterials markedly improve drug concentration at the lesion site and mitigate fibrosis, inflammation, and malignant tumor advancement by disrupting mechanical signaling pathways. The study clarifies their multifaceted benefits in treating COPD, IPF, and lung cancer, including decreased systemic toxicity and improved spatiotemporal control. Nonetheless, clinical translation continues to encounter obstacles, including impediments in large-scale production, inadequate compatibility with breathing devices, and disputes concerning long-term biosafety. In the future, the amalgamation of precision medicine, adaptive smart materials, and multi-omics artificial intelligence technologies will facilitate the development of individualized diagnostic and therapeutic systems, establishing a novel paradigm for the proactive management of respiratory disorders. This review offers essential theoretical foundations and technical approaches for the practical application of nanomaterials and the enhancement of therapeutic techniques in respiratory medicine.

## 1 Introduction

Respiratory diseases, such as chronic obstructive pulmonary disease (COPD), idiopathic pulmonary fibrosis (IPF), and lung cancer, impose a substantial global health burden by significantly impacting morbidity and mortality (Thai et al., 2021; Christenson et al., 2022; Liu G. Y. et al., 2022; Wu et al., 2023; Yao et al., 2023). Among these, COPD alone affects over 380 million individuals worldwide and ranks as one of the leading causes of death (Christenson et al., 2022), while IPF is a relentlessly progressive condition with limited treatment options and a median survival of only 3–5 years (Liu G. Y. et al., 2022). Lung cancer, meanwhile, is exceptionally lethal; data from 2024 indicate that it is responsible for more than 1.8 million deaths annually (Zhou et al., 2024) and that advanced cases have a 5-year survival rate below 10% (Bray et al., 2024). This highlights the critical demand for more effective treatment strategies (Thai et al., 2021). Additionally, the increasing prevalence of conditions such as asthma and acute lung injury underscores the critical need for effective prevention and management strategies for respiratory disorders (Shaw et al., 2021). An increasing body of evidence suggests that disturbances in the pulmonary mechanical microenvironment, a complex interplay of extracellular matrix (ECM) stiffness, alveolar surface tension, cellular forces, and cell-matrix interactions, play a crucial role in the pathogenesis and progression of these diseases (Liu H. et al., 2023). Excessive ECM stiffening triggers self-sustaining fibrotic cycles in IPF (Li et al., 2024), while altered mechanical forces underpin airway remodeling in COPD and support tumor growth and metastasis in lung cancer (Elbehairy et al., 2015). Overall, the dysregulation of the mechanical microenvironment represents a significant and multifaceted therapeutic target.

Owing to their unique size and highly tunable physicochemical properties, nanomaterials present unparalleled opportunities to finely adjust the pathological microenvironment within the lungs (Kinnear et al., 2017). Their ability to achieve targeted delivery [e.g., via specific cell-surface receptor binding (Kaneti et al., 2017)], controlled release kinetics [e.g., providing sustained drug exposure (Ehrmann et al., 2023)], and stimuli-responsiveness [e.g., activation by disease-specific cues such as reactive oxygen species (ROS) or pH (Liu et al., 2021)] presents innovative solutions to overcome the limitations of conventional therapies, including systemic toxicity and poor localization.

Here, we explore how nanomaterials can address dysregulation in the lung’s mechanical microenvironment by modulating ECM properties, normalizing alveolar surface tension, and influencing mechanotransduction pathways in pertinent lung cells. By evaluating recent advancements, we highlight potential of various nanostrategies, from targeted drug delivery systems to biomimetic constructs (Wang et al., 2020), to restore mechanical homeostasis in the lung. Furthermore, the review will discuss current challenges to the clinical translation of these nanotherapeutics, such as manufacturing scalability, integration with breathing devices, and long-term biosafety concerns. In summary, this review aims to systematically evaluate the design and application of nanomaterials in specifically targeting and modifying the impaired pulmonary mechanical microenvironment in COPD, IPF, and lung cancer.

## 2 Nanomaterial-driven therapies and mechanical regulation in respiratory diseases

**FIGURE 1:**
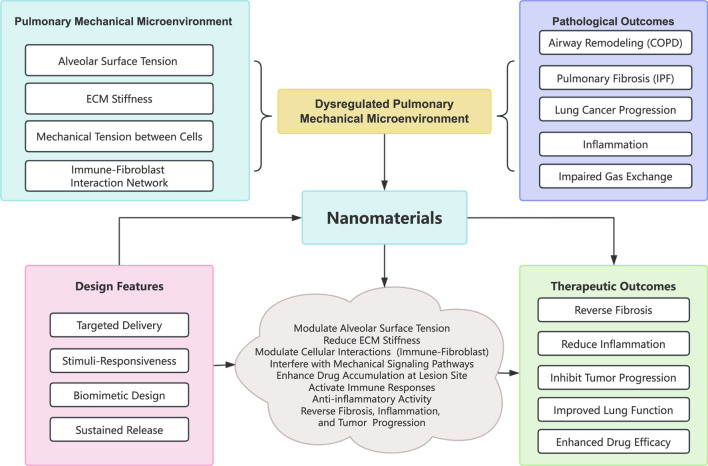
Conceptual diagram of the interplay between mechanical forces and nanomaterial interventions. Abbreviations: COPD, Chronic obstructive pulmonary disease; IPF, Idiopathic pulmonary fibrosis; ECM, Extracellular matrix.

In recent years, nanomaterials, especially metal-organic framework (MOF) materials like ZIF-8 nanoparticles, have shown remarkable promise in biomedicine, notably in treating respiratory diseases, due to their distinct versatility. Their fundamental modes of action include precisely targeting lesions, regulating drug release, activating immune responses, and interacting uniquely with cells and biomolecules (see Figure 1). ZIF-8 nanoparticles accumulate in diseased tissues, such as atherosclerotic ones, via the enhanced permeability and retention (EPR) effect. In doing so, they modulate lipid metabolism by activating autophagy and releasing anti-inflammatory agents, thereby reducing the disease severity (Sheng et al., 2022). Another crucial mechanism is the direct interaction of nanomaterials with cells. For instance, immunotherapy-based nanovaccines, constructed by combining redox-responsive antigen nanoparticles with TLR7/8 agonists, substantially enhance immune activation within lymph nodes (Wang et al., 2023). Additionally, nanomaterials can replicate both the structural and functional characteristics of viruses. For example, the nanovaccine IMDQ@OVA-TA, developed using a MOF template, achieves stability under physiological conditions through the molecular co-assembly of Zn^2+^-imidazole, Zn^2+^-tannic acid, and tannic acid bound to proteins. Its pH-sensitive coordination bonds facilitate controlled drug release, promote antigen escape from lysosomes, and enhance cross-presentation (Liu et al., 2024). These attributes render nanomaterials highly promising for various applications, including drug delivery, antibacterial and antiviral therapies, immunomodulation, and disease diagnosis. In addition, nanomaterials can be chemically modified to create MOF-Polyacrylonitrile (MOF-PAN) composite membranes, which capitalize on high hydrogen permeability and excellent separation performance, positioning them as strong candidates for gas separation applications (Li et al., 2014).

Nanomaterials demonstrate considerable potential in targeted therapies owing to their adjustable physicochemical properties, while the mechanical characteristics of biological matrices like lung ECM dictate cellular responses and overall tissue homeostasis. Seminal research on lung ECM mechanics has gradually revealed how the physical properties of the lung’s ECM, including stiffness, viscoelasticity, and non-linear responses to stretching, regulate cellular behavior and contribute to both lung homeostasis and the progression of disease. ECM stiffness acts not only as a structural component but also as a dynamic regulator of cellular processes in both healthy and diseased lung tissues (Guo, 2022). Notably, aging also induces significant changes in the biomechanical properties of the ECM at various lung volumes, which in turn impacts the survival and proliferation of mesenchymal stromal cells (Ulldemolins et al., 2024). Collectively, these studies underscore that the intrinsic mechanical properties of lung tissue are as crucial as biochemical signals for maintaining proper lung function.

In pulmonary fibrosis, several landmark studies have demonstrated that abnormal ECM mechanics play a central role in disease progression. For instance, baseline stiffness of ECM profoundly defines it non-linear response to cyclic stretching, thereby underscoring the matrix’s sensitivity to mechanical cues in fibrotic conditions (Júnior et al., 2023). Building on these insights, subsequent studies have also provided strong evidence that progressive ECM stiffening is closely linked to pathological remodeling in pulmonary fibrosis (Júnior et al., 2023; He et al., 2024). These conclusions are further supported by research on emphysema, where it has been described that disruptions in lung parenchymal mechanics can alter fibroblast activity (Leslie et al., 2021). In addition, it has been established that the dynamic interplay between ECM composition and cellular morphology plays a critical role in orchestrating inflammatory responses within the lung microenvironment (Hackett et al., 2022). Mechanical ventilation, a routine intervention in critical care, also serves as a model for studying ECM mechanics. Notably, this intervention can impair ECM integrity by modifying its structure and viscoelastic properties, potentially increasing the lung’s susceptibility to ventilator-induced injury (Al-Husinat et al., 2024). In a related investigation, quantifying the relaxation and creep responses of alveolar tissue has offered valuable insights into the lung’s mechanical resilience under both steady and stressed conditions (Beni et al., 2022).

Respiratory diseases are marked by intricate pathogenic mechanisms that encompass inflammatory responses, oxidative stress, ECM remodeling, apoptosis, and autophagy. These conditions are frequently associated with alterations in the pulmonary mechanical microenvironment, which comprises the composition and rigidity of the ECM, intercellular mechanical tension, and fluid shear stress. Changes in these parameters impact cellular activities such as proliferation, differentiation, migration, and apoptosis, thereby influencing disease progression. In IPF, elevated mechanical stress in type II alveolar epithelial cells and adverse interactions among myofibroblasts lead to excessive ECM deposition and structural lung damage (Li et al., 2024). Furthermore, dysregulation of the pulmonary microenvironment is linked to abnormal immune cell function. For instance, in the tumor microenvironment, tumor-associated macrophages (TAMs) can be reprogrammed into a pro-tumor M2 phenotype, which suppresses anti-tumor immune responses (Song et al., 2021). Changes in the mechanical microenvironment can also be converted into intracellular biochemical signals through mechanotransduction processes, further affecting cellular behavior and gene expression (Hong et al., 2024). In patients with asthma, alterations in zinc levels and its regulatory proteins in both the systemic circulation and lungs are closely associated with disease development, with zinc’s anti-inflammatory, antioxidant, and immunomodulatory properties making it a potential therapeutic target (Liu X. Y. et al., 2022). Many pulmonary diseases also involve microbial infections, such as bacterial pneumonia and viral infections like influenza and COVID-19. Bacteria can influence the speciation and migration of arsenic via redox reactions, playing a key role in the arsenic cycle, while selenium metabolism significantly impacts ecosystems through its role in selenoprotein biosynthesis and electron transport during respiration (Stolz et al., 2006). Additionally, cellular autophagy is vital in pulmonary diseases as evidenced by the observation that selenium inhibits intracellular *Mycobacterium tuberculosis* (MTB) by modulating macrophage autophagy-related pathways (Chen et al., 2021). Thus, a comprehensive understanding of the pathological mechanisms underlying respiratory diseases, particularly the role of the mechanical microenvironment, is crucial for developing innovative therapeutic strategies.

## 3 Nanomaterials in inflammation and the mechanical microenvironment

Nanomaterials show significant promise in modulating inflammatory responses and influencing the pulmonary mechanical microenvironment, offering novel strategies for respiratory disease treatment. They predominantly regulate inflammation through several mechanisms. First, as drug carriers, nanomaterials can facilitate the targeted delivery of anti-inflammatory medications to sites of inflammation, thereby enhancing local drug concentrations and efficacy while minimizing systemic side effects. For example, ZIF-8 nanoparticles loaded with losartan potassium can localize to inflammatory sites via the EPR effect, subsequently releasing anti-inflammatory agents (Sheng et al., 2022). Second, nanomaterials may possess inherent anti-inflammatory properties; selenium nanoparticles (SeNPs) considerably reduce early-stage inflammatory factor levels by alleviating alveolitis, general inflammation, and structural lung damage (Shahabi et al., 2021; Abdel-Wahhab et al., 2024). Studies indicate that SeNPs exert these effects by activating the Nrf2-Keap1 pathway to modulate selenoprotein expression (Qi et al., 2025). Additionally, nanomaterials can alter immune cell function: for instance, the ZIF-sealed Ti substrate glucose oxidase system (ZSTG) uses a “natural-artificial dual enzyme intervention (NADEI)” approach to improve a compromised immunological microenvironment and correct osteogenic dysfunction caused by implant-associated infections (Dong et al., 2024). On the other hand, PDGFB@ZIF8-RGD nanoplatform facilitates tumor vascular normalization and zinc ion-mediated immune activation, transforming a “cold” tumor immunological microenvironment into a “hot” one, thereby boosting anti-tumor immunity (Ma et al., 2024). These features underscore the versatility of nanoparticles for treating inflammation-related diseases (Li et al., 2024).

Nanomaterials also intervene in the pulmonary mechanical microenvironment through several pathways. First, they can modify the composition and structure of the ECM. For instance, cyclic RGDfC peptide-modified ZIF-8 nanoparticles (ZDFPR NPs) have been shown to effectively regulate the pulmonary mechanical microenvironment by reducing the mechanical tension in type II alveolar epithelial cells and inhibiting adverse interactions among myofibroblasts, thereby restoring mechanical homeostasis and arresting fibrotic progression (Li et al., 2024). Nanomaterials can also alter intercellular mechanical tension; secondary interactions between ZIF-8 nanoparticles and a polyurethane matrix have been found to significantly enhance the composite’s stiffness, fracture energy, and yield strength (Mahdi and Tan, 2016). Furthermore, nanomaterials can modulate cellular responses to mechanical cues by regulating intracellular signaling pathways. For instance, a multifunctional drug delivery system based on a C10-KR8 peptide combined with ZIF-8 nanoparticles promotes bone regeneration and reestablishes the immune microenvironment by modulating the Htra1/FAK/YAP pathway (Wang et al., 2024).

Particles with an aerodynamic diameter of around 1–3 μm are ideal for deep lung deposition, as they can maximize penetration into the alveolar region while minimizing impaction in the upper airways (Abdelaziz et al., 2020; Takeuchi et al., 2021). Nevertheless, particles within this size range are highly vulnerable to being cleared by alveolar macrophages, which can easily engulf particulates of micrometer size (Huang et al., 2024). One key way that nanomaterials avoid being engulfed by phagocytes is through their capacity to settle into the lung lining fluid without being immediately detected by these immune cells. For instance, polymeric nanocapsules with diameters of about 200 nm have been demonstrated to lodge within the lung lining fluid, where they stay until they dissolve. This allows them to temporarily escape both mucociliary clearance and clearance mediated by macrophages (Rodrigues et al., 2023). In contrast, particles with geometric sizes of around 1–2 μm are more easily recognized and engulfed by alveolar macrophages (Huang et al., 2024).

In addition, nanomaterials provide significant versatility through surface modification, which can further decrease phagocytic uptake. Surface alterations such as PEGylation or biomimetic coatings can prevent protein adsorption and opsonization, effectively “shielding” the nanoparticles from recognition by the mononuclear phagocyte system (Rampado et al., 2020; Mills et al., 2022). By doing so, these modifications allow nanoparticles to maintain their biodistribution and therapeutic payload within the lung tissue while evading rapid clearance. Moreover, studies have shown that nanoparticles can be engineered with specific physicochemical properties—such as optimal surface charge, hydrophilicity, and particle stability—that reduce interactions with immune cells, thereby increasing the likelihood that they will remain in the alveolar region (Kučuk et al., 2023).

Collectively, these studies underscore the significant potential of nanomaterials in targeting the pulmonary mechanical microenvironment, opening new avenues for respiratory disease treatment.

## 4 Clinical practice: from animal models to human application

Despite significant progress in fundamental research on nanomaterials for the treatment of respiratory diseases, their clinical application still faces numerous challenges. Translating these materials from laboratory studies to clinical practice requires overcoming obstacles related to safety, efficacy, large-scale production, quality control, and regulatory approval (see Table 1). Animal models remain indispensable in preclinical evaluations, providing critical insights into the efficacy and safety of nanomaterials. For example, in a C57BL/6 mouse model, nanovaccines demonstrated long-term accumulation at the injection site, sustained lymph node transport, and effective therapeutic outcomes against, E.G7-OVA lymphoma, thereby elucidating the mechanisms behind both innate and adaptive tumor-specific immune activation (Liu et al., 2024).

**TABLE 1:** Comparison of conventional therapies vs. nanomaterial approaches for respiratory disease.

Method	Conventional Therapies	Nanomaterial Strategies
Approaches (COPD)	Bronchodilators, Corticosteroids, Oxygen therapy, Pulmonary rehabilitation	Drug carriers for targeted delivery; Smart materials; ROS-sensitive nanocarriers
Approaches (IPF)	Antifibrotic drugs (Pirfenidone, Nintedanib), Oxygen therapy, Lung transplantation	Antifibrotic drug delivery; ROS-sensitive nanocarriers; Targeting ECM
Approaches (Lung cancer)	Surgery, Chemotherapy, Radiotherapy, Targeted therapies (small molecules, antibodies), Immunotherapy	Targeted chemotherapy; Biomimetic nanocarriers; pH-responsive materials; Gene therapy
Targeting Efficiency	Low, systemic distribution for COPD, IPF; Variable, targeted therapies improve but still have off-target effects for lung cancer.	High, designed for lesion-specific delivery
Specificity	Low, broad effects across the body for COPD, IPF; Variable, targeted therapies improve but still have off-target effects for lung cancer.	High, designed to target pulmonary microenvironment
Reported Adverse Events	Significant side effects (e.g., from corticosteroids, chemotherapy, radiotherapy)	Potentially lower systemic toxicity; Controversies regarding long-term biosafety
Cost	Relatively lower cost, widely available, generic options for COPD; Relatively high cost for antifibrotics, transplant costly in IPF; High cost for targeted therapies and immunotherapy in lung cancer.	Potentially higher cost, especially in early stages
Scalability	High, well-established production and distribution for COPD; High for drugs, low for transplantation in IPF; Variable, targeted therapies and immunotherapy are complex in lung cancer.	Bottlenecks in industrial-scale production, inhalation device compatibility challenges
Complexity of Production	Low, well-established manufacturing processes for COPD; Medium for drugs, high for transplantation in IPF; High for targeted therapies and immunotherapy in lung cancer.	High, more complex manufacturing processes, nanotechnology expertise needed

Abbreviations: COPD, Chronic obstructive pulmonary disease; IPF, Idiopathic pulmonary fibrosis; ECM, Extracellular matrix; ROS, reactive oxygen species.

Current clinical trials on nanotherapy for respiratory diseases mainly focus on using nanocarriers to deliver therapeutic agents or siRNA for targeted treatment of specific pathologies (see Table 2). For instance, trial NCT03819387, a Phase I study for non-small cell lung cancer (NSCLC), employs lipid nanoparticles (LNPs) to deliver siRNA targeting glutathione S-transferase P (GSTP) with the goal of inhibiting tumor growth in KRAS-mutated cancers. Additionally, trial NCT03608631 investigates the exosome-mediated delivery of KRAS G12D siRNA for pancreatic cancer, which, although not directly focused on respiratory diseases, highlights the broader therapeutic potential of nanocarriers in oncology (Narasipura et al., 2023). Furthermore, preclinical research has explored the nanoformulation of glucocorticoids (e.g., dexamethasone) or antioxidants (e.g., N-acetylcysteine) using liposomal or polymeric nanoparticles to treat pulmonary inflammation; however, these strategies remain in the early stages of development (Yu and Qiu, 2024).

**TABLE 2:** List of nanomaterials used for pulmonary drug delivery.

Nanomaterial	Key properties	Advantages	Primary applications
Liposomes	Spherical phospholipid bilayers; tunable size/charge; PEGylation possible	High biocompatibility; encapsulates hydrophilic/hydrophobic drugs; passive/active targeting; protects payload	NSCLC, pulmonary infections, gene therapy (mRNA), COPD
Solid Lipid Nanoparticles (SLNs)	Solid lipid matrix; physiological lipids; crystalline structure	High physical stability; controlled release; avoids premature drug expulsion; minimal toxicity	Chronic diseases (COPD, asthma); prolonged drug exposure
Nanostructured Lipid Carriers (NLCs)	Blend of solid + liquid lipids; disordered structure	Higher drug loading than SLNs; reduced drug expulsion; surface-functionalized	Tuberculosis, lung cancer, targeted delivery to alveolar macrophages
Nanoemulsions	Oil-in-water droplets; submicron size; self-emulsifying	Solubilizes lipophilic drugs; rapid absorption; easy nebulization; high bioavailability	Lipophilic drug delivery (antifungals, antivirals)
Microstructured Lipid Carriers (MLCs)	Hybrid solid lipid + oil matrix; disordered internal structure	Enhanced drug retention; improved stability; prolonged release; reduced β-modification risks	Delicate therapeutics (peptides, proteins); chronic fibrosis
Mesoporous Silica Nanoparticles	Inorganic; high surface area; tunable pore size	High drug loading; controlled release; surface modifiable	Co-delivery of drugs/imaging agents; lung cancer theranostics
Surfactant-Coated Nanogels	Hybrid polymer-lipid matrix; coated with lung surfactants	Enhanced mucus penetration; protects nucleic acids; improves transfection efficiency	Gene therapy (siRNA, mRNA); pulmonary fibrosis; cystic fibrosis

Abbreviations: COPD, Chronic obstructive pulmonary disease; SLN, Solid lipid nanoparticle; NLC, Nanostructured lipid carrier; MLC, Microstructured lipid carrier; NSCLC, Non-small cell lung cancer; PEG, Polyethylene glycol.

Polymeric nanoparticles and lipid-based delivery systems, such as liposomes, solid lipid nanoparticles (SLNs), and nanostructured lipid carriers (NLCs), provide controlled drug release and improved bioavailability. These are particularly beneficial for conditions, such as, acute respiratory distress syndrome (ARDS) and other inflammatory pulmonary disorders, where traditional treatments often fail to achieve sufficient concentrations at the target site without causing systemic toxicity (Yu and Qiu, 2024). Targeted nanotherapies have demonstrated effectiveness in NSCLC, a leading respiratory malignancy. For example, polymeric nanoparticle-based treatments, engineered to target the epidermal growth factor receptor (EGFR), which is often overexpressed in NSCLC, deliver therapeutic agents directly to cancer cells, enhancing efficacy while minimizing side effects (Crintea et al., 2023). Similarly, lipid-based nanoparticles demonstrate key advantages such as enhanced formulation stability, ease of scale-up, and opportunities for surface modification that enable site-specific targeting. These systems are capable of encapsulating both hydrophobic and hydrophilic drugs, making them versatile for delivering a wide spectrum of therapeutics to lung tissues, and their ability to penetrate the pulmonary mucus barrier is critical for effective drug absorption in the lung (Kumari et al., 2020). Additionally, advancements in anti-inflammatory nanotherapies have broadened treatment options for respiratory diseases. In this context, bioactive cyclodextrin-based nanoparticles demonstrate the ability to neutralize ROS and regulate the activity of inflammatory cells (Li et al., 2023). By mitigating oxidative stress and reducing inflammation, these therapies show promise for diseases such as COPD and ARDS, while also enabling localized treatment within the pulmonary system and minimizing the systemic side effects typically associated with conventional anti-inflammatory drugs (Li et al., 2020).

Incorporating vibrating mesh atomizers into spray drying systems has significantly improved the production of submicron particles. Recent studies indicate that combining vibrating mesh technology with spray drying generates uniform lactose-leucine microparticles that are well-suited for pulmonary delivery. This approach harnesses rapid evaporation kinetics and precise droplet formation to generate particles with optimal aerodynamic profiles and improved deposition characteristics (Thiyagarajan et al., 2021). Furthermore, the improved evaporation of droplets produced by vibrating mesh atomizers results in a more consistent drying process, which minimizes particle size variability and complies with rigorous pharmaceutical quality standards (Thiyagarajan et al., 2021).

Comparative studies of nebulizer technologies indicate that vibrating mesh devices deliver more precise control over droplet size and produce a gentler aerosolization process compared to traditional jet and ultrasonic nebulizers (Ashraf et al., 2020). Additionally, the successful application of vibrating mesh atomizers in clinical settings, such as in the delivery of inhaled ribavirin, demonstrates their practical benefits in preserving drug stability and maintaining therapeutic efficacy during both aerosolization and subsequent drying processes (Dallas et al., 2020). Further improvements in vibrating mesh design, including increased pore uniformity and durability, have extended their use beyond aerosol generation for inhalation therapy to broader roles in pharmaceutical drying, where producing submicron particles is critical for optimal drug delivery performance (Puig et al., 2023). AeroVanc^®^, a dry powder inhaler formulation that encapsulates vancomycin in nanoparticles, has completed clinical trials for treating methicillin-resistant *Staphylococcus aureus* (MRSA) lung infections in cystic fibrosis patients (Waterer et al., 2020). Although AeroVanc^®^ effectively reduced MRSA burden, its DPI design required careful optimization to ensure uniform nanoparticle dispersion and consistent lung deposition. Surface modification of nanoparticles with hydrophilic polymers (e.g., polyethylene glycol) or lipid coatings has been shown to enhance aerodynamic properties by reducing aggregation during nebulization. Moreover, optimizing particle size distribution (e.g., 100–200 nm) and incorporating excipients like leucine has been found to improve aerosol stability and promote deep lung penetration (Zheng et al., 2024). 

Nebulization offers clear benefits for the pulmonary delivery of hydrophobic drugs by directly aerosolizing liquid nanocrystal suspensions. This technique eliminates the need for excipient carriers, thereby reducing formulation complexity and preserving the intrinsic properties of the drug (Lokugamage et al., 2021). It has shown particular efficacy in delivering biologics, as demonstrated by the effective nebulization of Coenzyme Q10 nanocrystals (Sun et al., 2024). Nevertheless, the inherent physicochemical instability of nanocrystalline suspensions during storage necessitates formulation adjustments. Operational factors, such as the type of nebulizer (jet, ultrasonic, or vibrating mesh) and various process parameters, can significantly influence aerosol generation efficiency, requiring device-specific optimization. These challenges underscore the need for advanced stabilization techniques in liquid-based nanocrystal delivery systems (Cuccia et al., 2023; Huang et al., 2025). In contrast, DPIs offer enhanced thermodynamic stability by immobilizing nanocrystals on engineered carriers through processes like spray-drying or lyophilization (Chang et al., 2021). The carrier matrix in these systems serves a dual purpose: it facilitates the redispersion of nanocrystals upon aerosolization and optimizes pulmonary deposition through tailored aerodynamic design (B et al., 2019). Although the propellant-free operation of DPIs enables patient-driven administration with a lower environmental impact, formulation challenges remain. It is crucial to precisely control carrier-nanocrystal interfacial interactions to balance adhesion strength with payload release kinetics (Li et al., 2025). Moreover, process-induced nanocrystal aggregation often necessitates the inclusion of dispersion enhancers, further adding to formulation complexity. The multi-stage production process, encompassing nanocrystal micronization, carrier blending, and drying, also presents scalability issues, highlighting the need for cost-effective manufacturing innovations.

The therapeutic potential of nanomaterials in reshaping the pulmonary mechanical microenvironment ultimately depends on resolving inherent batch-to-batch variability, a major regulatory hurdle for respiratory applications (Mülhopt et al., 2018). Variations in synthesis parameters (such as precursor ratios and temperature ramping rates) and purification efficiency can directly influence nanomaterial stiffness, surface topology, and subsequent mechanobiological interactions with lung tissue. To establish robust structure-activity relationships in pulmonary mechanotherapy, it is essential to employ multi-platform characterization strategies: (1) triangulated size analysis using transmission electron microscopy (TEM) with AI-enhanced particle segmentation, scanning mobility particle sizer with electrostatic spectrometry (SMPS-ES), and dynamic light scattering (DLS) to assess agglomeration states affecting pulmonary deposition; (2) high-resolution TEM/scanning electron microscopy (HRTEM/SEM) for quantifying primary particle crystallinity and aspect ratios, which determine nanomaterial-induced alveolar strain modulation; and (3) screening for trace metal contaminants via inductively coupled plasma mass spectrometry (ICP-MS) or optical emission spectrometry (OES), as these may alter bronchial epithelial mechanosensing. Notably, SMPS-ES has been shown to outperform DLS in reproducibility for dry-powder-derived nanomaterial aerosols that require controlled reagglomeration, an important prerequisite for uniform engagement with the air-blood barrier. Establishing standardized protocols that align synthesis variables with the mechanical properties of nanomaterials is a priority to meet the requirements set by regulatory agencies such as the Food and Drug Administration (FDA) and the European Medicines Agency (EMA) for inhalable nanoformulations. This analytical framework is key to developing clinically relevant nanomaterial designs that can predictably modulate pulmonary viscoelasticity while simultaneously mitigating immunomechanical risks.

## 5 Biosafety concerns in pulmonary nanotherapeutics

Nanomaterials can be engineered with tunable size, surface chemistry, and composition, enabling them to effectively navigate the lung’s intricate environment and improve therapeutic outcomes in conditions such as COPD and acute lung injury (Alotaiq and Dermawan, 2024). However, these very characteristics can also provoke unforeseen toxicological interactions, thereby necessitating a rigorous evaluation of their biosafety profiles. While innovations in nanocarrier design, such as controlled drug release and targeted delivery, hold considerable promise, concerns remain about oxidative stress, inflammatory responses, and immunogenicity arising from nanoparticle interactions with lung tissues (Chehelgerdi et al., 2023; Sun et al., 2023). Multiple studies have indicated potential toxicity and the risk of long-term negative effects on lung health (Zhao et al., 2023; Feng et al., 2024; Wen, 2024). This dual nature underscores the importance of adopting a balanced approach that optimizes therapeutic benefits while ensuring safety in pulmonary nanomedicine.

One notable biosafety concern involves MOFs, especially zeolitic imidazolate frameworks such as ZIF-8. Under physiological conditions, the inherent instability of ZIF-8 can result in the leaching of zinc ions (Zn^2+^), which disrupts cellular homeostasis and triggers oxidative stress in lung epithelial cells (Sun et al., 2023). Excess Zn^2+^ disrupts mitochondrial function, triggers pro-inflammatory cytokine release, and may initiate apoptotic pathways, particularly in patients with pre-existing lung damage (Chehelgerdi et al., 2023). Variations in pH and interactions with alveolar fluids can amplify ion release, highlighting the necessity for precise control over nanoparticle degradation kinetics (Alotaiq and Dermawan, 2024). Even at “non-cytotoxic” doses, ZIF-8 nanoparticles can increase endothelial permeability by inducing actin reorganization and disrupting cell junctions in human aortic endothelial cells (Liu J. Y. et al., 2023). The potential cytotoxicity of ZIF-8 derivatives largely arises from Zn^2+^ overload and ROS generation. In in vitro endothelial barrier assays, more than 50 μM Zn^2+^ derived from ZIF-8 degradation reduced transendothelial electrical resistance (TEER) by 40%, activating MAPK/NF-κB pathways that increased vascular permeability (Liu Z. et al., 2022). In contrast, controlled release formulations, such as SF-coated MN patches, helped mitigate toxicity by maintaining 85% cell viability in L929 fibroblasts even after a 72-h exposure (Liu Z. et al., 2022). Efforts to reduce Zn^2+^ leaching primarily focus on material engineering strategies. For example, surface functionalization with biocompatible polymers (e.g., polyethylene glycol) or lipid coatings can lower ion diffusion rates while still preserving drug encapsulation efficiency (Sun et al., 2023). Additionally, partially substituting zinc with more biocompatible metals (such as magnesium or calcium) within the MOF structure can stabilize the framework and diminish toxic ion release (Chehelgerdi et al., 2023). Despite these advances, a trade-off between maintaining structural integrity and achieving optimal therapeutic performance remains a challenge. For example, while organic nanocarriers like liposomes typically exhibit lower metal toxicity, they often do not match the drug-loading capacity and stability offered by ZIF-8 (Al-Ghamdi et al., 2022). Thus, continuous design refinements, underpinned by advanced *in vitro* and *in vivo* models, are crucial for achieving a balance between efficacy and safety.

Regulatory bodies such as the FDA and EMA have implemented rigorous guidelines to address the specific challenges encountered with pulmonary nanotherapeutics, ensuring comprehensive preclinical evaluations including pharmacokinetics, biodistribution, and toxicity studies, with particular attention to lung-specific endpoints like alveolar inflammation and mucociliary clearance (Alotaiq and Dermawan, 2024). For metal-based systems like ZIF-8, a rigorous evaluation of ion leaching kinetics and the resulting cellular damage is essential, often using advanced techniques such as ICP-MS to quantify Zn^2+^ release (Sun et al., 2023). The FDA’s evolving framework now incorporates computational tools, such as physiologically based pharmacokinetic (PBPK) modeling, to forecast nanoparticle behavior in the lung and assess risks associated with particle aggregation or metal ion release (Al-Ghamdi et al., 2022). Similarly, the EMA stresses the importance of thorough physicochemical characterization, such as stability studies under simulated physiological conditions, to ensure consistency across production batches (Chehelgerdi et al., 2023). Both agencies advocate for a “safety-by-design” approach, encouraging proactive modifications-such as surface coatings or ion substitution-to preempt toxicity during early development phases (Alotaiq and Dermawan, 2024). Ensuring product safety throughout its lifecycle depends on comprehensive post-market surveillance and adherence to quality-by-design principles. Regulatory guidelines require manufacturers to conduct rigorous in-process monitoring during scale-up to guarantee consistent nanoparticle uniformity and to minimize variations in critical parameters such as size and ion release rates (Sun et al., 2023). Moreover, adaptive clinical trial designs facilitate the collection of real-world safety data, enabling swift responses to address emerging risks, such as immunogenicity in susceptible populations (Al-Ghamdi et al., 2022).

It should be noted that there is a lack of systematic validation of the dynamic intervention capability and long-term safety of nanomaterials in the lung mechanics microenvironment. Existing studies mainly focus on the static mechanical properties (e.g., elastic modulus) of nanomaterials, while neglecting their structural stability and functional maintenance ability under dynamic stresses of the respiratory cycle. The drug release kinetics of drug-carrying nanoparticles may be significantly altered during alveolar expansion-contraction, but there is a lack of corresponding *in vitro* bionic models for evaluation. In addition, the effects of long-term retention of nanomaterials on the mechanical properties of lung tissues (e.g., local stress redistribution, extracellular matrix remodeling) have not been elucidated, and the potential risks include progression of fibrosis or impairment of airway epithelial barrier function. Existing safety evaluations are mostly based on acute toxicity experiments, and long-term tracking studies of the cumulative effects of nanomaterial degradation products in the mechanical microenvironment are lacking.

## 6 Future perspectives: from precision medicine to smart materials

Nanotechnology has transformed medicine by enabling the development of advanced drug delivery platforms and personalized therapeutic strategies. Current advancements use nanoscale materials to improve therapeutic indices, minimize side effects, and enhance targeting specificity and overall efficacy. In the realm of precision medicine, where treatments are tailored to individual patient profiles, these innovative nanoformulations are increasingly indispensable, as they can be precisely engineered to match each patient’s distinct pathophysiological characteristics (Napione, 2021; Zhong et al., 2021; Feng et al., 2024). Moreover, recent progress in nanotechnology has resulted in the development of smart materials capable of responsive drug delivery. These smart nanomaterials are engineered to release therapeutic agents in response to environmental triggers such as pH changes, temperature fluctuations, or specific biomolecular interactions. This targeted release enhances treatment efficacy while minimizing the off-target effects associated with conventional delivery systems (Palmieri et al., 2020; Feng et al., 2024). However, the inherent complexity of manufacturing nano-drug delivery systems poses challenges in maintaining consistent quality across different batches. Although nanocarriers like liposomes and polymer micelles have shown significant efficacy in clinical trials, the complexity of their production often results in considerable batch-to-batch variation, which can affect their clinical viability (Zheng et al., 2025). Consequently, numerous technical challenges remain in scaling up the production of nanotechnology-based smart materials from the laboratory to industrial settings. The integration of artificial intelligence (AI) and machine learning with nanotechnology platforms represents a key advancement in personalized medicine, as these technologies enable the design of treatment regimens that adapt to ongoing patient responses and allow for timely modifications to therapy protocols (Lopez-Cantu et al., 2022; Rawat et al., 2022; Mittal et al., 2024).

Machine learning, in particular, aids in accurately predicting interactions between nanomaterials and biological systems, a critical factor when developing inhalable products (Feng et al., 2024). Models that combine molecular fingerprints with graph neural networks have demonstrated high accuracy in forecasting nanomaterial toxicity and interactions, thereby facilitating the rapid screening and optimization of candidates with therapeutic potential while minimizing cytotoxicity (Ji et al., 2022; Dhoble et al., 2024). Such approaches offer a more rational design strategy that accelerates discovery by reducing the reliance on costly and time-consuming *in vitro* and *in vivo* testing. However, integrating AI into nanomedicine for diagnostics and treatment also brings complexity. Advanced algorithms and data processing are needed, and the resulting complexity can hinder patient comprehension of the underlying concepts and associated risks. This issue can complicate informed consent, as patients may not fully understand the potential effects of the treatment. Additionally, nanomedicine involves the aggregation, storage, and use of substantial amounts of personal health data for AI model development, which raises concerns about data privacy, unauthorized access or misuse of this data would constitute a breach of privacy (Blackman and Veerapen, 2025).

The potential applications of nanotechnology in respiratory diseases extend to real-time diagnostics as well. Nanosensors and nanoimaging techniques enable the early detection of respiratory conditions by identifying biomarkers at the nanoscale, offering sensitivity and specificity beyond what conventional methods provide (Palmieri et al., 2020; Zhong et al., 2021; Prabhakar et al., 2023; Feng et al., 2024). In the context of personalized medicine, these advances empower healthcare providers to develop treatment strategies tailored to the unique biological signatures of patients’ respiratory conditions, thereby enhancing therapeutic effectiveness. Moreover, the collaboration between nanotechnology and traditional medical approaches highlights priority areas for clinical translation toward integrative health solutions. For example, combining traditional herbal formulations with nanocarriers can improve the bioavailability and targeted delivery of herbal compounds, addressing the longstanding issues of poor solubility and limited bioavailability encountered in many traditional medicines (Rc, 2022; Bojanić et al., 2023). However, this hybrid approach necessitates rigorous evaluation of the interactions between nanomaterials and natural compounds, along with their combined safety profiles (Rc, 2022; Sharma and Sahu, 2023). However, existing delivery systems have limited spatiotemporal control of drug release, making it difficult to achieve site-specific responsive release at the focal site. Meanwhile, the lack of universality of nanocarrier surface modification strategies makes it difficult to take into account the physicochemical properties of different drugs and the heterogeneous needs of lung tissues. Together, these factors constrain the translational application of nano-delivery systems in the treatment of lung diseases.

Our work uniquely focuses on nanomaterials’ role in modulating the pulmonary mechanical microenvironment (ECM stiffness, surface tension, cellular forces). From our perspective, as clinicians and researchers acutely aware of the limitations in current respiratory disease management, the potential of nanomaterials to reshape the pulmonary mechanical microenvironment represents a transformative frontier. Although recent preclinical data compellingly demonstrate targeted action and reduced systemic toxicity, significant challenges remain on the path to routine clinical use, challenges that include scalable manufacturing, device compatibility, long-term biosafety assurance, and regulatory navigation. Nonetheless, we firmly believe that the targeted, stimuli-responsive, and biomimetic properties of nanotherapeutics hold the key to overcoming current therapeutic ceilings in diseases such as COPD, IPF, and lung cancer. It is imperative for clinicians to stay informed of these rapid advancements, as they signify a shift from merely managing symptoms or slowing disease progression to actively intervening at the mechanobiological level. Looking forward, while nanotechnology offers a promising future for transforming therapeutic landscapes in respiratory diseases, notable challenges, particularly relating to safety, efficacy, and regulatory compliance, must be addressed. Continued interdisciplinary collaboration is essential to bridge the gap between exciting benchtop discoveries and tangible bedside applications, ultimately fulfilling the promise of personalized nanomedicine for respiratory patients.

## 7 Conclusion

Nanomaterials, a pivotal technology transforming the management of respiratory diseases, are creating novel avenues to tackle significant clinical issues such as COPD, IPF, and lung cancer, by precisely modulating the pulmonary mechanical microenvironment. Their targeted delivery, stimuli-responsive, and biomimetic design characteristics not only address the limitations of conventional therapies, such as elevated systemic toxicity and inadequate lesion targeting, but also offer multifaceted approaches for reversing the malignant progression of diseases by modulating alveolar surface tension, ECM stiffness, and the immune-fibroblast interaction network. In the future, the profound integration of precision medicine and smart materials, alongside multi-omics technologies, artificial intelligence, and dynamically responsive nanoplatforms, will facilitate the development of personalized diagnostic and therapeutic systems, effecting priority areas for clinical translation from “passive treatment” to “active regulation.” The clinical translation of nanomaterials must address fundamental challenges, including large-scale production, biosafety assessment, and the optimization of inhalation devices. This necessitates interdisciplinary collaboration, enhancement of standardized evaluation systems, and robust interaction between fundamental research and clinical applications. This approach will maximize the promise of nanotechnology while maintaining safety, transforming the future of respiratory disease therapy.
